# Exploring perception and usage of narrative medicine by physician specialty: a qualitative analysis

**DOI:** 10.1186/s13010-021-00106-w

**Published:** 2021-10-20

**Authors:** Daniel A. Fox, Joshua M. Hauser

**Affiliations:** 1 Northwestern University Feinberg School of Medicine, Chicago, IL USA; 2 Section of Palliative Care, Northwestern University Feinberg School of Medicine, Chicago, IL USA

**Keywords:** Narrative Medicine, Physicians, Specialty, Medical Education

## Abstract

**Background:**

Narrative medicine is a well-recognized and respected approach to care. It is now found in medical school curricula and widely implemented in practice. However, there has been no analysis of the perception and usage of narrative medicine across different medical specialties and whether there may be unique recommendations for implementation based upon specialty. The aims of this study were to explore these gaps in research.

**Methods:**

Fifteen senior physicians who specialize in internal medicine, pediatrics, or surgery (5 physicians from each specialty) were interviewed in a semi-structured format about the utilization, benefits, drawbacks (i.e., negative consequences), and roles pertaining to narrative medicine. Qualitative content analysis of each interview was then performed.

**Results:**

Three themes emerged from our analysis: roles, practice, and outcomes. Through these themes we examined the importance, utilization, barriers, benefits, and drawbacks of narrative medicine. There was consensus that narrative medicine is an important tool in primary care. Primary care physicians (general internists and general pediatricians) also believed that narrative medicine is not as important for non-primary care providers. However, non-primary care providers (surgeons) generally believed narrative medicine is valuable in their practice as well. Within specialties, providers’ choice of language varied when trying to obtain patients’ narratives, but choice in when to practice narrative medicine did not differ greatly. Among specialties, there was more variability regarding when to practice narrative medicine and what barriers were present. Primary care physicians primarily described barriers to eliciting a patient’s narrative to involve trust and emotional readiness, while surgeons primarily described factors involving logistics and patient data as barriers to obtaining patients’ narratives. There was broad agreement among specialties regarding the benefits and drawbacks of narrative medicine.

**Conclusions:**

This study sheds light on the shared and unique beliefs in different specialties about narrative medicine. It prompts important discussion around topics such as the stereotypes physicians may hold about their peers and concerns about time management. These data provide some possible ideas for crafting narrative medicine education specific to specialties as well as future directions of study.

**Supplementary Information:**

The online version contains supplementary material available at 10.1186/s13010-021-00106-w.

## Background

### Narrative medicine

While there is no consensus definition of narrative medicine [1], Rita Charon, who coined the term, defined it as practicing medicine with narrative competence [2]. Narrative competence is “the ability to acknowledge, absorb, interpret, and act on the stories and plights of others” and requires using textual skills, creative skills, and affective skills when reading or listening to these stories to successfully achieve [2, 3].

Narrative medicine has been found to strengthen the relationships physicians have with colleagues [2], to promote empathy [2, 4], and to aid in the healing of patients [4, 5] and family [6]. However, experts have also noted that narrative medicine may be difficult to learn, and there is a risk that physicians who practice it may disregard other important components of patient care [5, 7].

### Implementation

In the 20 years since it was first defined [6], narrative medicine has become a well-known and respected approach to care. It has been implemented in a vast range of ways and formally taught in health systems across the world. For example, *My Life, My Story*, an oral history project in the Department of Veterans’ Affairs (VA) collects abbreviated life stories of patients for placement in their electronic medical records for providers to access [8]. Numerous guides exist to help teach the practice of narrative medicine [4, 7]. Recently, the Association of American Medical Colleges (AAMC) put out a call for narratives from health care providers in light of the COVID-19 pandemic [9]. There is programming and courses at medical schools and in graduate medical education specifically committed to teaching narrative medicine [10–13].

**Fig. 1:**
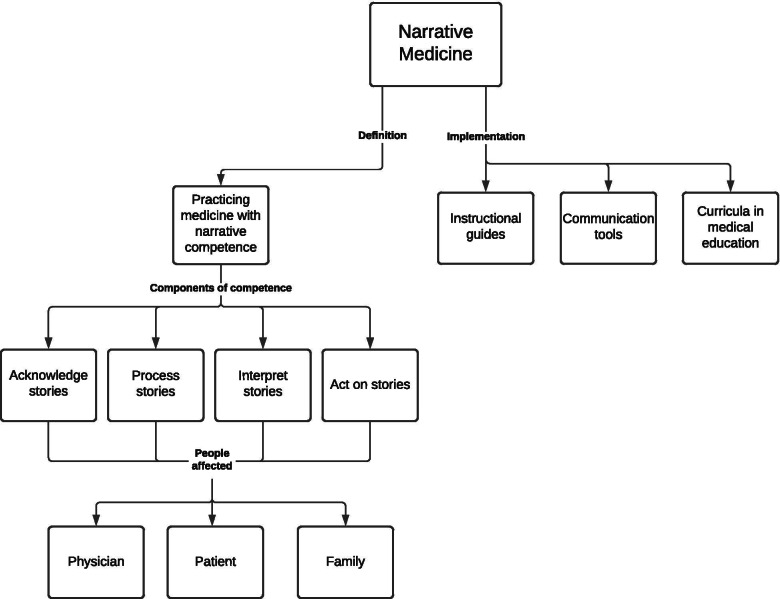
Conceptual model of narrative medicine. Note—The definition of narrative medicine shown is based upon the one provided by Charon (2001)

Figure 1 is a conceptual model of narrative medicine that unifies the definition described above along with forms of implementation.

### Perspectives

A number of studies have been conducted analyzing patients’ and family caregivers’ perspectives on narrative medicine [14] as well as those of health care providers who have been through a formal narrative medicine intervention [10–13]. Huang and colleagues found significant differences in the perception of narrative medicine in students learning Western medicine compared to those learning Chinese medicine, suggesting cultural and philosophical differences within training and practice may lead to a difference in perception and usage of narrative medicine [15]. Analogous to the distinctions between Western and Chinese medicine, medical specialties may develop their own unique cultures [16–19] which may lead to different perceptions and approaches to care, such as a person-centered mindset frequently seen in family physicians [20] or a “fix it” mentality often found in surgeons [21].

Despite the increasing implementation of narrative medicine in medical school curricula and calls for implementation into practice, there has been no analysis of the perception and usage of narrative medicine across different medical specialties and whether there may be unique recommendations for implementation based upon specialty. This study aimed to explore these perceptions and usages of narrative medicine as well as possible modes of implementation by performing a qualitative study of physicians from different specialties.

## Methods

### Study setting and design

From August through October 2019, physicians from 3 different specialties at Northwestern Medicine (internal medicine, pediatrics, and surgery), a multi-specialty group based at Northwestern University Feinberg School of Medicine were interviewed by a study team member and completed a form collecting demographic information following the interview. The interviews were semi-structured, contained 16–25 questions depending on interview trajectory, and lasted 15–30 min each. The interview script, including definitions (Additional file 1), was developed by study team members in consultation with an expert in narrative medicine and two physician stakeholders. All interviews were audio-recorded and later transcribed.

Domains of questions included utilization, benefits (with specific questions about decision-making), drawbacks (i.e., negative consequences), and roles regarding narrative medicine. The interview included reading a sample story taken with permission from the *My Life, My Story* veterans’ oral history project at William S. Middleton Memorial Veterans Hospital [8] and then gauging the participant’s opinion. Additionally, there were 5 questions in the interview exclusively for pediatricians. The data collected from the oral history and pediatric-specific questions will be analyzed and discussed in future publications.

### Participants

Fifteen senior physicians at Northwestern Medicine who specialize in internal medicine, pediatrics, or surgery (5 physicians from each specialty) were enrolled. This sample size reached theoretical saturation, the point at which additional data would not further develop any category or theme found in analysis [22].

### Recruitment

Potential participants were directly identified by study team members based on eligibility to participate per the inclusion criteria. Participants were recruited via personal invitation via email. Participants were purposefully sampled [23] to ensure diversity of experience and clinical exposure (Table 1).

**Table 1:** Participant Demographics

n = 15	#	Male (n)	Average years in practice (range)	Percent of Job in clinical practice (range)	Average length of new patient appointment in minutes (range)	Average length of established patient appointment in minutes (range)
Internists	5	2	20 (5—26)	81% (50—100)	44 (40—60)	22 (20 – 30)
Pediatricians	5	1	12.8 (3—27)	75% (20—100)	21 (10—45)	12.5 (10 – 15)
Surgeons	5	4	17 (7—40)	82% (70—100)	28 (20—30)	14 (10 – 15)

### Statistical analysis

Qualitative content analysis of each interview transcript was performed [24]. Both study team members, who have experience with qualitative analysis and backgrounds in medical humanities, used an inductive coding approach during their independent coding of transcripts. A coding schema was established through constant comparison [22, 25], in order to further develop codes throughout the analysis process. Following the independent coding of each transcript, coders would meet to resolve any discrepancies from independently coding. Once the initial round of coding was complete, all transcripts were re-analyzed to ensure the complete capture of data in relation to the developed coding schema. Final categories and themes were then identified. We used *NVivo* software (QSR, Version 12) to store and organize our data.

## Results

Three themes emerged from our analysis: roles, practice, and outcomes. We define roles as the roles and responsibilities physicians identify for themselves and others in the practice of narrative medicine. We define practice as how narrative medicine is used (or not used) by physicians. We define outcomes as the opinions held by physicians regarding the consequences of using narrative medicine. Within these themes, we examined the importance, utilization, barriers, benefits, and drawbacks of narrative medicine.

### Roles in narrative medicine: who should use it?

There was consensus among physicians from all three specialties that narrative medicine is important within primary care, which typically was attributed to the importance of the longitudinal relationships primary care physicians have with patients (Table 2).

**Table 2:** Physicians’ beliefs about roles in narrative medicine

Description	Representative Quotes
Consensus that narrative medicine is important within primary care	*“I think* [narrative medicine]*’s a very important part of the getting to know someone, and especially in a field, like primary care when the expectation is I’m gonna know them for years if not decades.” (Internist)*
	*“I can imagine discussing this with an internist let’s say and ‘well their personal conditions has dramatic effect on how I’m going to manage their diabetes because how they spend their time outside these office visits kind of dictate what I think about their glucose control.’” (Surgeon)*
Most surgeons believe narrative medicine is important for their practice	[In response to practicing narrative medicine]*“…it’s very important that there is trust in the relationship early on…I think that connecting with them personally helps foster that trusting relationship which I think is important in all physician relationships, but it's important it's established quickly in a surgical relationship with a surgeon.” (Surgeon)*
	*"If I meet them initially and they’re with their spouse we will often have a conversation about their, their lives together. If they have a surgery and they’re in the hospital, we have a conversation about their recovery often with their family and what their expectations are for their recovery. That often brings in things about their life story" (Surgeon)*
Primary care physicians generally view narrative medicine as less important in other specialties	*“You know, does every doctor have to know their life story?…You know if you’re just having a knee replacement maybe you don’t need to know” (Internist)*
	*“I can see when someone comes in to the* [Emergency Room]*…they’re not gonna want me to be sitting there for half an hour asking them about what you know their whole history and stuff like that. So I do think…what type of care you’re providing matters, and how that would impact the relationship and the embracement of narrative medicine.” (Pediatrician)*

Most surgeons also believed narrative medicine was important for their own practice (Table 2), with one surgeon notably dissenting: *“…you want to hear that knowing someone's life story is going to change something, but surgery is just one of those peculiar specialties where again if somebody comes to see with a surgical problem that can only be treated surgically, a lot of times they're kind of prepared for that before the first office visit… It's different than if I had a longer time, term relationship with the patient.”*

In contrast to the surgeons’ beliefs about their practice, primary care physicians in our sample identified non-primary care specialties as placing less value on narrative medicine (Table 2).

### Practice of narrative medicine: how is it used?

Most pediatricians and surgeons agreed that the severity and/or complexity of the problem mattered when determining which patients for which to utilize narrative medicine; they were more likely to employ narrative approaches in the setting of complex and serious illness. There was more division among internists regarding whether the seriousness of the illness mattered or not.

There was diversity within all specialties about what wording to use with patients when eliciting a patient’s illness narrative (Table 3). Internists described eliciting a patient’s narrative over a number of visits, while surgeons generally collect the narrative in the first visit (Table 3). Pediatricians were divided along this point, with one making mention of how it depends on their patient: *“…I could see you know in example of the teen I might be getting a little bit of a larger life story the first time I meet them. But then in the example of a newborn or a younger child, I’m initially just getting a regular social history and then as time progresses more often, only if there are issues would I be asking the parent about the larger life story.”*

**Table 3:** Physicians’ beliefs about the practice of narrative medicine

Description	Representative Quotes
Division within all specialties about what wording to use with patients	*“If I'm in the clinic I try to make it more directed, kind of like you know, ‘I'm interested in getting to know you as a patient and a person. I’d love for you to tell me something about your life’ or ‘I’d love to tell me little bit about you, not from being a patient’” (Internist)*
	*“I don’t think that I explicitly do, what I typically try and find* [the patient’s narrative] *out more indirectly through the course of responses to more standard questions that I would ask related to their past medical history so if there are things that come up related to changing jobs or moving, but I don't typically try set that out as a specific part of their narrative.” (Internist)*
Internists generally collect a patient's narrative over time	*“So I can collect this kind of information over periods of visits and I remember things about patients. You know I’ve had patients for 25 years…and you get little things every time you see somebody… I start to know these kind of details but would I get all of this in one sitting, just the way that medicine is practiced right now? That would be difficult.” (Internist)*
	*"…as I look at my own patients and the ones that I’ve had really great longitudinal relationships with, I think some of that naturally comes out of getting to know someone over time." (Internist)*
Surgeons generally collect a patient's narrative in the initial visit	*“…in that initial visit I'll ask all patients what they do for a living. I allow other elements of personal life to come out just in the flow of conversation, but I definitely try to elicit specific personal information so that I can…just allow personal details to dictate the flow of the interview as it, as it proceeds.” (Surgeon)*
	[In response to when the narrative is collected] *"Oh it would be at the first office* [visit] *because the, I’ll tell you that most of the time I see a patient one time before their operation, and one time after their operation." (Surgeon)*
Factors involving the patient's emotional readiness were often described as barriers to collecting the narrative by primary care physicians	*“Sometimes, if it's the first time I’m meeting them there isn't that level of trust where they’re gonna tell me everything right away, and in my particular case, there are some things parents don't want to share about their past in front of their child.” (Pediatrician)*
	*“I’ll occasionally run into someone who wasn't quite expecting* [collecting the patient’s narrative]*. Maybe they aren’t looking for that in their relationship with their doctor, so they are kind of maybe either off-put or kind of surprised by the question, but for the most part I feel like that doesn't happen.” (Internist)*
Factors involving logistics and patient data were often described as barriers to collecting the narrative by surgeons	*“Well time is part of it, and I don't know how to better say that, but meeting a patient in clinic and wanting to understand the life story to sort of get focused and relatively concise assessment or feeling like I understand their life a little better within the time constraints…I think that’s the biggest barrier” (Surgeon)*
	*"…then you're essentially relying on the family to tell their story which obviously may not be as the patient would tell story to you." (Surgeon)*

Barriers to collecting the narrative that involved the patient’s emotional readiness were described more often by primary care physicians (Table 3). Pediatricians in particular saw trust as a large barrier to obtaining a patient’s narrative, while internists were more likely to note a patient’s psychosocial factors (e.g., cultural identity, previous trauma) as a barrier.

Barriers involving logistics or patient data were more frequently noted by surgeons (Table 3). Specifically, time was seen as a barrier by most surgeons, as well as half of the primary care physicians interviewed.

A minor but notable category brought up is the intuition involved in utilizing narrative medicine. Two primary care physicians described that narrative medicine is intuitive for them. From a pediatrician: *“It just seems like humanism and things like that. It always strikes me as that's what were supposed to do as a doctor, right?”* In contrast, one surgeon noted that narrative medicine had to be deliberately learned over the course of years: *“I learned that maybe 10 or 15 years ago […] to step out of acting as a doctor and just be a person talking to him in the office, so I try to be with them in the offices as I am out of the street with anybody else.”*

### Outcomes of narrative medicine: what are the benefits and drawbacks?

When describing the benefits of narrative medicine, there was consensus that obtaining a patient’s narrative is emotionally positive for physicians. There also was consensus that obtaining a patient’s narrative is beneficial for the doctor-patient relationship across multiple domains, including connection, communication, empathy, and trust (Table 4).

**Table 4:** Physicians’ beliefs about the outcomes of narrative medicine

Description	Representative Quotes
Consensus that narrative medicine is emotionally positive for physicians	*“So I think it always benefits me to hear anyone’s life story even if, I mean even on a personal level if I’m not taking care of the patient, it’s always enriching to learn about what someone else's been through.” (Pediatrician)*
	*"I take a lot of like pleasure in getting to know patients over a long period of time…their perspective on the lives…has benefited me just to think about that in the context of my life for my family members lives, that's sort of thing. Sort of gives me a sense of appreciation I guess, gratitude for life." (Surgeon)*
Consensus that narrative medicine benefits the doctor-patient relationship along multiple spectrums	*“I know things about my patients that have nothing to do with their care and that just makes me feel like there's a real connection, and it's just humanizing all around. So I think that this is a really important part of how you really develop that trust because you also value their story right?” (Internist)*
	*"…* [the patient’s narrative] *helps me I guess* [be] *a little bit more…sensitive to her anxiety about an upcoming operation beyond the usual anxiety that…a patient has" (Surgeon)*
Consensus that narrative medicine aides in decision-making	*“…if somebody grew up in an environment where they were always told that western medicine is bad…A medication…that I prescribed for their child…I might prescribe it and talk about it and they might nod, say sure and go home and not use it. It would be much better for me to know their attitude about it coming in. It’s just gonna affect how I handle it, my decision-making” (Pediatrician)*
	*“I mean I always think that you need to know a lot about somebody to try to figure out how to help treat them…Like knowing all sorts of details about how they approach life and how they approach medicine…does affect treatment plans so you have to kind of take all of that into account.” (Internist)*
Consensus that narrative medicine can be emotionally negative for physicians	*“There’s a lot of bad life stories out there that are hard, so it makes you feel bad, but uh…usually I’d rather know them than not know them, but yeah it’s more emotionally draining.” (Internist)*
	*“Definitely hearing about trauma that patients have experienced has affected me emotionally and I kind of carry it with me throughout the rest of the day or the rest of the week, or forever, sometimes just thinking about it” (Pediatrician)*
Consensus that there are logistical and data-driven drawbacks to narrative medicine	*“I mean the biggest drawback is time right? We would all love to spend an hour talking to every single patient because people are fascinating and people do have very interesting life stories” (Surgeon)*
	*“Well sometimes you can over interpret something, you can go to the other extreme, you know, you think you know a person well and…sometimes you just take a shortcut and assume they’re either doing this is because of what happened back then, but if you don't clarify it with them you might misinterpret it or over interpret something” (Pediatrician)*

There was near consensus that narrative medicine helps with decision-making (Table 4). More miscellaneous informative benefits (coordination of care, informing care of future patients, etc.) were discussed, but none of these were mentioned by a majority of physicians or majority within a specialty.

When describing the drawbacks of narrative medicine, there was consensus that obtaining a patient’s narrative can be emotionally negative for physicians (e.g., “emotionally draining”). There also was consensus that there are logistical and data-driven drawbacks to obtaining a patient’s narrative (e.g., it takes too much time, it elicits distracting information) (Table 4). A minority of physicians from each specialty also noted that obtaining a patient’s narrative can be emotionally negative for the patients. For example, a pediatrician noted eliciting a narrative in one of their patients may have triggered traumatic memories: *“I wonder if it did like trigger some like PTSD…even though I knew much more about the situation.”*

## Discussion

### Do beliefs about narrative medicine reflect stereotypes?

This study found there was a general consensus that narrative medicine is important within primary care. Most surgeons agreed that narrative medicine is important in their practice too, but most primary care physicians thought it was less important in specialties outside of primary care. Conventional stereotypes held by health care providers about surgeons include that they are less “warm” (e.g., not patient-oriented, aggressive, etc.) than other physicians [26–28]. Although such stereotypes may not be accurate, it may be that primary care physicians are reflecting these stereotypes about surgeons, contributing to the perception that narrative medicine is less important outside of primary care.

It is notable that when discussing barriers to collecting a patient’s narrative, surgeons were more likely to describe barriers involving practical aspects (such as logistics and time) rather than barriers involving a patient’s emotional readiness that were primarily described by primary care physicians.

While these beliefs may align with stereotypes, it is also important to consider that these beliefs could be attributed to other factors, such as workflow. In addition, non-primary care specialties include a wide range of specialties beyond surgeons, suggesting the opinions primary care providers hold may be due to in-group bias [29] rather than holding stereotypes about surgeons. Further research might investigate primary care physicians’ underlying beliefs as to why they see narrative medicine as less valuable in other specialties.

### Primary care consensus of beliefs about the analyzed themes of narrative medicine

Pediatricians and internists expressed similar beliefs in all themes analyzed more often than either specialty did with surgeons. This divide suggests unique programming for narrative medicine might be crafted for primary care specialties compared to other specialties. This is particularly true when emphasizing the importance of narrative medicine within primary care and when describing circumventions to the barriers of obtaining a patient’s narrative due to their emotional readiness, as these were two places in the data where internists and pediatricians were in agreement.

### Consensus on outcomes

All three specialties were in general agreement regarding the benefits and drawbacks of narrative medicine. This is instructive for it suggests that when creating programming for narrative medicine, particularly for graduate medical education, instructors do not need to formulate any specific appeals for the use of narrative medicine based on the specialty being taught.

### The ideal strategy for collecting narratives depends on workflow and preferences

Our data reveal that internists were more likely to collect patients’ narratives over the course of many visits compared to surgeons, who more often described collecting patients’ narratives in the first visit. Thus, for those who establish longer term relationships with their patients (e.g., internists) it seems practical to elicit the narrative over the course of many visits, while physicians who provide more acute care (e.g., many surgeons or hospital medicine physicians) might prioritize getting as much of the narrative as possible in the first visit.

The specific wording physicians use with patients when collecting their narratives varied within all specialties, suggesting this is a matter of personal preference and comfort rather than a distinction based on specialty workflow, culture, or education.

These data indicate narrative medicine programming should consider the workflow of the specialty when discussing the timeframe for collecting narratives and that the wording used with patients can vary based on what feels most comfortable to the physician, within certain parameters.

### The challenges of time: both a barrier and drawback of narrative medicine

Physicians in our sample described time as both an example of a logistical barrier to collecting narratives and as a practical drawback of narrative medicine. This aligns with known worries of physicians. Time management has been described as a major concern of physicians leading to numerous negative outcomes, including physician dissatisfaction [30, 31], patient dissatisfaction [32, 33], and limited shared decision-making [32, 33]. Possible solutions to these challenges include simultaneously educating physicians on time management strategies [34, 35] during narrative medicine programming or exploring more time-efficient strategies to elicit the patient’s narrative.

### Study limitations

There is reasonable concern we primed participants on the value of narrative medicine for decision-making by prompting them to discuss the subject during the interview (Additional file 1). Consequently, there should be skepticism that narrative medicine aiding decision-making is truly a near consensus benefit. However, decision-making is a critical component of patient interaction, and contextual considerations, like a patient’s narrative, are an often-overlooked component of decision-making [36]. Additionally, as noted in our conceptual model (Fig. 1), a component of narrative medicine is acting on stories, an action that intrinsically involves decision-making. As such, our team decided it was appropriate to explicitly prompt participants on this topic.

Another limitation is that our participants were not necessarily expected to have nuanced views of narrative medicine and thus were provided a definition curtailed for that aspect for the sake of efficiency (Additional file 1), again noting this abbreviated definition was approved by an expert in narrative medicine. Some may argue this definition is too simplistic and does not capture the full depth of the term. It is worth asking whether, had we provided a more detailed definition, physicians’ responses would have changed and whether our definition led physicians to provide too limited or too broad of responses. This limitation lends value to formulating a universal definition for narrative medicine [1].

Finally, participants were all affiliated with one academic medical center in a single metropolitan area. It is worth considering these participants may hold different beliefs regarding narrative medicine than physicians who are unaffiliated or located in diverse geographic regions. Further studies with more robust demographic diversity of employers and location asking these same questions are warranted.

Despite these limitations, this study produced a rich set of data that can guide both physician education and future directions of study on narrative medicine.

## Conclusion

This study reveals several shared and unique beliefs regarding narrative medicine held by different specialists. The shared beliefs included the importance of narrative medicine within primary care, the benefits of narrative medicine, and its drawbacks. The areas where there were more diverse perspectives included narrative medicine’s importance for providers outside of primary care, the barriers to collecting narratives, and strategies for collecting narratives.

These data provide a wealth of suggestions for focusing narrative medicine programming by specialty and future directions of study. As this burgeoning field of medicine continues to grow, these focused questions on how to adapt narrative medicine to different physicians’ practices become all the more relevant.

## Supplementary Information

**Additional file 1**. Interview Script. Script used for interviewing participants.

## Data Availability

The datasets generated and/or analyzed during the current study are not publicly available due to privacy considerations but are available from the corresponding author on reasonable request.

## Abbreviations

VA: Veterans’ Affairs
AAMC: Association of American Medical Colleges
