# Supplementary material for: Exploring perception and usage of narrative medicine by physician specialty: a qualitative analysis
Source: Philos Ethics Humanit Med. 2021 Oct 20;16:7. doi: 10.1186/s13010-021-00106-w (PMC8526278; doi:10.1186/s13010-021-00106-w)
Supplement: Supplementary file 1 — Additional file 1. Interview Script. Script used for interviewing participants. [file 13010_2021_106_MOESM1_ESM.docx]

**Narrative Approaches to Care Interview Questions and Script**

This interview will last roughly 20 minutes. You do not have to answer any questions you do not

want to. You may also stop the interview or take a break at any time. Is it ok if we get started?

First, I will provide you with one definition of narrative approaches to care, the topic of this study. It places a premium on understanding a patient’s larger life story.

1. Do you ever explicitly ask the patient to tell their larger life story?
   1. [If yes] Tell me more about how you go about doing so.
   2. [if no] Why not?
   3. What challenges have you had around getting their story?
   4. When do you learn their larger life story in your time caring for them? [potential probes: at beginning of relationship; when faced with a serious illness; when faced with an important clinical decision]
2. How do other providers in your practice (e.g. NPs, PAs) contribute to obtaining a patient’s larger life story?
3. Are there ways in which the patient’s larger life story affects decision-making?
4. What do you think are the benefits, if any, of taking into account patients’ larger life story?
   1. Could you provide me an example of a time when taking into account a patient’s larger life story benefited a patient?
   2. Could you tell me if and how it has ever benefited you? [probe if necessary: for example, benefitted around clinical decision or intervention, obtaining diagnostic information]
5. What do you think are the drawbacks or downsides, if any, when caring for a patient, taking into account their larger life story?
   1. Could you provide me an example of a time when taking into account a patient’s larger life story had a negative effect on a patient?
   2. Could you tell me if and how it has ever had a negative effect on you?
   3. How could taking into account a patient’s larger life story be improved?
6. Do you find your feelings about taking into account a patient’s larger life story are consistent or do they change based on context? i.e. Are there situations/times in which you believe taking into account a patient’s larger life story is more or less important?
7. PEDS ONLY: How important is it to take into account parents’ larger life story, both as a couple and individuals?
8. PEDS ONLY: How important is it to take into account a child’s larger life story?
   1. Do parents’ and child’s larger life stories interplay with each other when considering treatment, care, etc.?
   2. At what point does the child begin to construct their own life story in a way that is important in your interactions with him/her?
9. Do you believe there are any [other] unique considerations when taking into account a patient’s larger life story in your specialty or your particular practice style?
10. I am going to provide you a sample story from a project using a narrative approach to care called *My Life, My Story*, an oral history project that originally began at William S. Middleton VA in Madison, WI. Volunteers collect Veterans’ oral histories and write up a 1000-word summary that are put in their EMR for their providers to access.
    1. What are the first things that jump out at you after reading this?
    2. Say this was your patient, how could this help guide their care?
    3. Having discussed this, does this change any of your answers to the previous questions?
11. Is there anything else about this topic that you’d like to add?

Thank you for taking the time to talk with me today.
